# Markerless Human Motion Tracking Using Hierarchical Multi-Swarm Cooperative Particle Swarm Optimization

**DOI:** 10.1371/journal.pone.0127833

**Published:** 2015-05-15

**Authors:** Sanjay Saini, Nordin Zakaria, Dayang Rohaya Awang Rambli, Suziah Sulaiman

**Affiliations:** Computer and Information Science Department, Universiti Teknologi PETRONAS, Tronoh, Perak, Malaysia; University of Minnesota, UNITED STATES

## Abstract

The high-dimensional search space involved in markerless full-body articulated human motion tracking from multiple-views video sequences has led to a number of solutions based on metaheuristics, the most recent form of which is Particle Swarm Optimization (PSO). However, the classical PSO suffers from premature convergence and it is trapped easily into local optima, significantly affecting the tracking accuracy. To overcome these drawbacks, we have developed a method for the problem based on Hierarchical Multi-Swarm Cooperative Particle Swarm Optimization (H-MCPSO). The tracking problem is formulated as a non-linear 34-dimensional function optimization problem where the fitness function quantifies the difference between the observed image and a projection of the model configuration. Both the silhouette and edge likelihoods are used in the fitness function. Experiments using Brown and HumanEva-II dataset demonstrated that H-MCPSO performance is better than two leading alternative approaches—Annealed Particle Filter (APF) and Hierarchical Particle Swarm Optimization (HPSO). Further, the proposed tracking method is capable of automatic initialization and self-recovery from temporary tracking failures. Comprehensive experimental results are presented to support the claims.

## Introduction

Markerless articulated human motion tracking is an emerging field with potential applications in areas such as automatic smart security surveillance [1], medical rehabilitation [2], and 3D animation industries [3]. The primary objective of markerless articulated human motion tracking is to automatically localize the pose and position of a subject from a video stream (sequences of images). A dominant line of approaches to the task is one that utilizes a 3D articulated human body model. The key idea is to render the body model and to compare the rendered image with acquired video frame in order to determine the fitness of the body model configuration. The optimization problem then becomes that of determining the body model configuration which will result in the best match to the images in the video. The key challenge in the approach is the high-dimensionality of the search space involved, due to the large number of degrees of freedom (DOF) typically present in an articulated human body figure. Other challenges include cluttered background, occlusion, ambiguity and illumination changes.

Many solutions have been proposed for model-based articulated human motion tracking. Until recently, most recent work are based on variants of local optimization method such as particle filtering (PF) [4–8]. To tackle the high-dimensionality of the problem, some solutions partition the search space [9, 10] while some others utilize multiple stage search operation [4, 7]. Most of the solutions based on local optimization however suffer from the curse of dimensionality and rely on simple human models (which lead to suboptimal tracking results) or require a high number of evaluations to provide satisfactory results.

More recently, stochastic global optimization methods such as the population-based evolutionary algorithms (EAs) [11] and swarm intelligence (SI) [12–16] have been gaining popularity. These methods have the ability to approximate highly non-linear problem, with relatively robust and reliable performance, and with relatively fewer tuning parameters [17–19]. Particle Swarm Optimization (PSO) [12, 13], in particular, has been becoming popular in human motion tracking. PSO, unlike PF, allows particles to interact with one another, and the interactions has been shown to be effective in finding global optima in high-dimensional search space.

The PSO delivers good average performances and is relatively easier to implement. In spite of its reported success however, the major issue in using PSO for articulated human motion tracking problem is that of particle diversity loss [18]. Generally, it occurs due to the convergence of the current optimization to the prior solution. All particles may be close to the previous optimum position; in other words, the swarm has shrunk. The swarm may still be able to find the optimum if the position of the new optimum lies within the region of the shrinking swarm. However, the true optimum may never be found if the current optimum lies outside of the swarm. Particles then are considered to be trapped in local minima. Hence, in a dynamic optimization problem such as tracking, it is necessary to control the particle diversity within the swarm at every temporal step.

In order to tackle this problem, in the context of model-based articulated motion tracking, in this paper, we have proposed what is to be referred to as Hierarchical Multi-swarm Cooperative Particle Swarm Optimization (H-MCPSO). Multiple swarms coordinate with little extra computation cost to find the optima in the articulated human motion tracking search space. In fact, the main contribution in this paper can be stated as follows: A novel hierarchical multi-swarm cooperative particle-swarm optimization method that combines several strategies to track full-body articulated human motion from multi-view video sequences. A comprehensive experimental evaluation of H-MCPSO along with the state-of-the-art methods, namely APF and HPSO, using the Brown and the HumanEvaII dataset, pointed to the superiority of the proposed approach.

## Related Work

### PF-based approaches

To address human motion tracking challenges, many approaches have been proposed in the literature. Earlier approaches include Particle Filters (PF). In particular, the condensation algorithm [8] has been widely used in human motion tracking. However, PF has shortcomings, namely computational cost, slowness of convergence, the curse of dimensionality and the need to rely on simple human models (which lead to sub-optimal tracking results) or the need for a high number of evaluations [17–20].

To address the issue, Annealed Particle Filter (APF) was introduced in [4]. APF merges condensation and simulates annealing in an attempt to improve the tracking results as well to reduce the number of particles. The APF performs a multilayer particle evaluation, where the fitness function in the initial layers is smoothed to avoid the search from being trapped in local minima. In the last layers, the fitness function is “sharpened” in order to concentrate the particles to solution regions.

Partitioned sampling (PS) [9] is another approach to address the dimensionality issue of PF. The complete search space is partitioned into several subsets (“partitions”). Consecutively, the dynamics for every partition is computed followed by a weighted resampling procedure. The technique was initially introduced in [21] to address the high cost effect of particle filters while tracking multiple objects.

The main problem with PS is in determining the optimal partitioning. In an attempt to solve this, in [10], a method was proposed that combines both PS and APF. The APF is incorporated into a PS framework by utilizing an appropriate weighted resampling in each sub-space. This approach is able to deal with high dimensionality but it suffers from the high cost of employing a very large number of evaluations per frame (around 8000).

The work in [5] focussed on improving the tracking accuracy and on reducing the computational cost of PF. It proposed a progressive particle filter where the mean shift strategy [22] is combined with a standard particle filter and a hierarchical search. The approach has however only been tested with single camera video sequences from a non-public dataset. It is not clear whether the approach would work on multi-view video sequences.

In [7], a multi-layer tracking framework was designed that combines stochastic optimization, filtering, and local optimization. In the first layer, pose was estimated using a global stochastic optimization method called interacting simulated annealing (ISA) and in the second layer, the estimated pose was refined by filtering and local optimization. Although the approach was shown to be capable of generating good tracking results, the main drawback of their approach is that it is not likely that the method can be extended to higher DOF because of the high number of particles required which leads to high computational cost.

In [23], a detailed comparison of stochastic PF algorithms for articulated body tracking was presented. The comparative study indicated that stochastic methods are more accurate than deterministic methods. However, stochastic methods are computationally heavy, especially in high dimensional search space.

### Evolutionary Computation Approaches

Over the past decades, the field of global optimization has been very active and various evolutionary algorithms have been proposed for solving a wide range of continuous optimization problem in science and engineering [24]. However, there has been few reported applications of global optimization to model-based articulated human motion tracking problem.

In [25], PSO was applied for upper body pose estimation from multi-view video sequences. The PSO algorithm was applied in a 20-dimensional search space. The optimization process was executed over 6 hierarchical steps based on the model hierarchy. However, the approach was used only to estimate static upper body pose. In a latter work [26], the number of optimized parameters was iteratively increased so that a superset of the previously optimized parameters is optimized at every hierarchical stage.

In [17], a hierarchical particle swarm optimization (HPSO) algorithm was presented for full articulated body tracking in multi-view video sequences. In order to overcome the high dimensionality problem, the 31-dimensional parameter search space was divided into 12 hierarchical sub-spaces. The approach was claimed to outperform PF, APF and a PS-APF hybrid. However, the shortcoming of the approach is that the HPSO algorithm optimization is unable to escape from local maxima which is calculated in the previous hierarchical levels. Moreover, the final solution tends to drift away from the true pose, especially at low frame rates.

In [27], a global local PSO (GLPSO) method was introduced for 3D human motion capture. The system reported divided the entire optimization cycle into two parts; the first part estimated the configuration for the whole body, and the second refined the local limbs poses using smaller number of particles. A similar approach called global-local annealed PSO (GLAPSO) was presented in [28]. In [28] however, the algorithm maintains a pool of candidates instead of selecting only the global best particle, in order to improve its search ability. Furthering the work, in [29], a resampling method was used to select a record of the best particle according to the weights of particles making up the swarm. This resampling leads to the reduction of premature stagnation.

In most approaches, hard partitioning of the search space was deployed, that is, a subset of parameters are optimized while the rest of the parameters are fixed. However, hard partitioning leads to error accumulation [20]. The error accumulation occurs due to that the fitness function for a particular stage cannot be evaluated completely independently from that for the subsequent stages. In mitigation of the problem, in [20], a soft partitioning approach was deployed with PSO (SPPSO). In the approach, the optimization process was divided into two stages; in the first stage, important parameters (typically torso) were optimized, and in second stage, all the remaining parameters were optimized while at the same time the estimates from the first stage are refined. Due to the use of global optimization, the approach is computationally expensive and its convergence slows down considerably near the global optimum when applied to a high-dimensional parameter search space.

Various combination of PSO approaches with other techniques such as dimensionality reduction and subspace have also been reported to address the human pose tracking problem. In [30] for example, a hybrid generative-discriminative approach was introduced for markerless human motion capture using charting and manifold constrained PSO. The charting algorithm has been used to learn the common motion in a low-dimensional latent search space and the pose tracking is executed by the modified PSO. In [31], a generative method for articulated human motion tracking using sequential annealed particle swarm optimization (SAPSO) was proposed. Simulated annealing principle has been integrated into traditional PSO to derive a global optimization solution. The main novelty of the approach was the use of Principal Component Analysis (PCA) to reduce the dimensionality of the problem and to learn the latent space of human motions. In spite of their sophistication and reported successes however, the approaches in [30] and [31] both rely on sequence-specific motion model, that is, they can only track pre-learned motions.

## Particle Swarm Optimization

PSO is a population-based stochastic optimization algorithm inspired by the way in which a crowd of birds or fish moves towards predefined target. The algorithm maintains a swarm where each particle represents a candidate solution to the optimization problem under consideration, and assumes the dimensionality of the search space involved.

The PSO is initialized with a set of *N* random particles, *x*_*i*_ (0 ≤ *i* < *N*). A cost (fitness) function measures the fitness of each of the particles. The fitness value is calculated by an observation model and the velocity provides the direction of particle movement. In each iteration, the movement of the *i*th particle depends on two key factors: its individual best position, *p*_*i*_, and second, the global best position, *g*, that is the best position that has been attained by the particles in the entire swarm.

For each iteration *t*+1, each particle updates its position and velocity according to the following equation:

$$\begin{array}{c} v_{t+1}^{i}=\omega v_{t}^{i}+\varphi_{1}r_{1}\left(p_{t}^{i}-x_{t}^{i}\right)+\varphi_{2}r_{2}\left(g_{t}-x_{t}^{i}\right) \end{array}$$ (1)

$$\begin{array}{c} x_{t+1}^{i}=x_{t}^{i}+v_{t+1}^{i} \end{array}$$ (2)

In Eq 1, $v_{t}^{i}$ and $x_{t}^{i}$ denotes the velocity vector and the position vector of particle *x*_*i*_ respectively at iteration *t*. The particle velocity is used to control the particle movement in search space and is useful when attempting to balance between exploitation and exploration. $p_{t}^{i}$ is the best-fitness position visited so far by the particles and *g*_*t*_ is the global best-fitness position visited so far by any particle of the swarm. *φ*_1_ and *φ*_2_, represent the positive constants known as cognitive and social parameters respectively. Both control the balance of influence between the personal best and the global best particle position. *r*_1_ and *r*_2_ are random numbers obtained from a uniform distribution within the interval [0, 1]. *ω* is an inertia weight parameter that functions as a velocity constraint mechanism [12]. It plays an important role in controlling the trade-off between global and local search. A higher *ω* value promotes particles that explore in large space (global search) whereas a smaller value encourages particles to search in smaller volumes (local search). Typically, to balance between global and local search, the inertia value is initialized to be high (*ω* = *ω*_*max*_) and as the search runs, it is gradually decreased down to the minimum (*ω* = *ω*_*min*_).

## Proposed H-MCPSO algorithm

The main drawback of PSO is premature convergence when applied to a high-dimensional parametric search space such as that in the pose tracking problem. Generally, the learning of each swarm particle is driven by the global best, *gbest*, even if the current *gbest* is not the global optimum. As a consequence of this, the particles may increasingly tend to be trapped in local optima as the number of degrees of freedom increases. Our proposed solution to overcome this problem as well as to increase general search efficiency in high dimensional parametric search space, is what we have been refering to as H-MCPSO (Hierarchical Multi-Swarm Cooperative Particle Swarm Optimization). In the proposed approach, a population is split into multiple sub-swarms and a master swarm. As the evolution progresses, new promising particles are delivered by the slaves to the master swarm. The master swarm evolves on its own term using the particles supplied by the slaves and its own. A symbiotic relationship is maintained between the master swarm and sub-swarms, which enhances the co-evolution and maintain a suitable diversity in the population.

Co-evolutionary paradigm in multi-swarm PSO can be broadly classified into two main categories, namely competitive co-evolution [32, 33] and cooperative co-evolution [32, 34, 35]. For the former, the subpopulations compete to gain an advantage over the others. For the latter, the subpopulations exchange information during the evolutionary process. In competitive co-evolution, ideally each and every particles from the competing subswarms compete with every other particles to determine the extent of its dominance. However, such an exhaustive approach requires extensive computational effort and is practically infeasible. Both cooperation and competition can in fact be combined into a single scheme as illustrated in [33].

**Fig 1:**
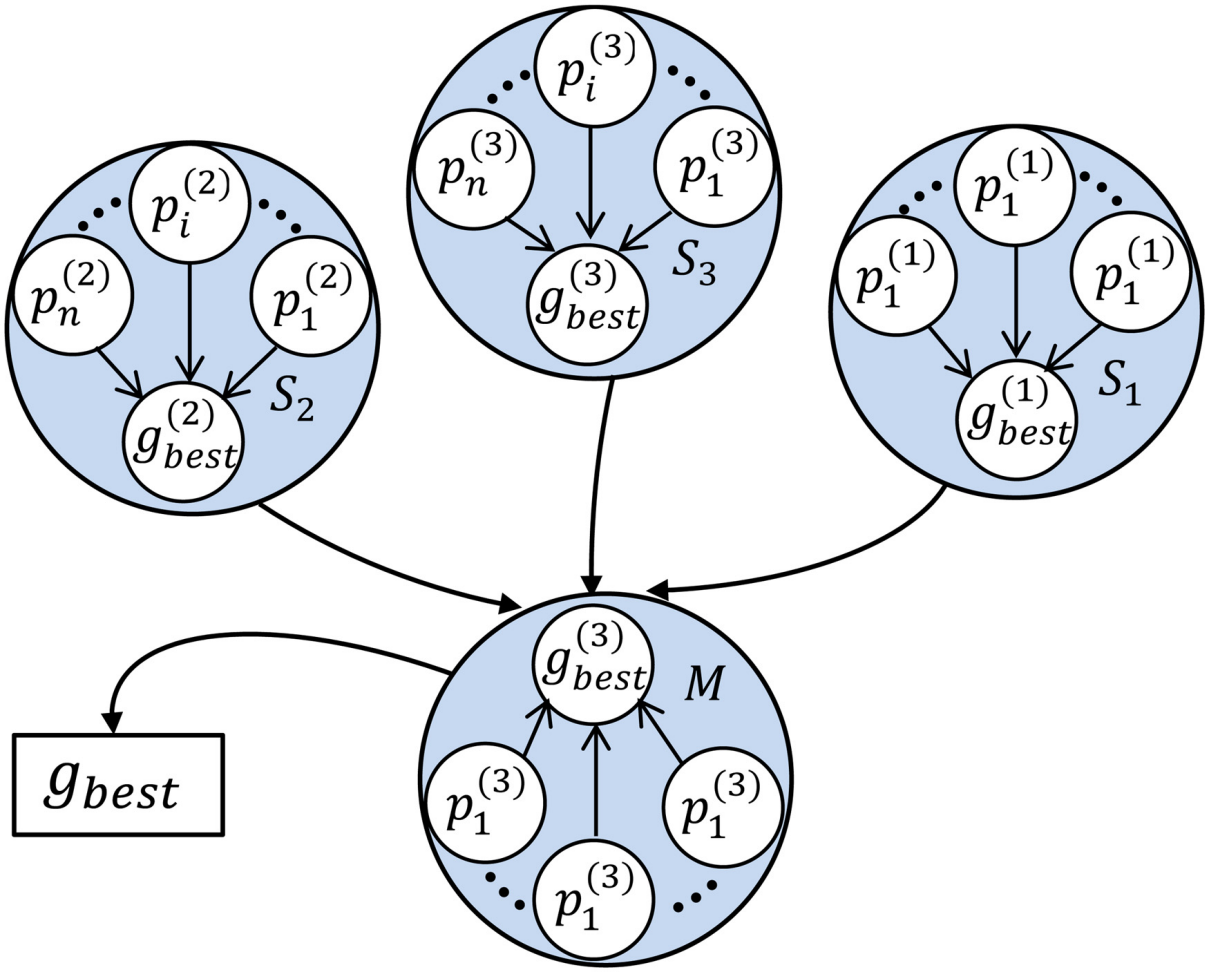
Illustration of Master-subswarm Communication Model.

In this paper, a cooperative co-evolutionary process has been used, because it seems to provide sufficient accuracy in our experiments and is simple to implement. Fig 1, illustrates the relationship between the sub-swarms and the master swarm and the communication model used for swarm synchronization.

As shown in Fig 1, a single PSO is executed by each sub-swarm. At the end of each iteration, each sub-swarm sends its best individuals to the master swarm. The master swarm selects the best of all received individuals and evolves on its own term and advances according to the equations below:

$$\begin{array}{ccc} v_{i}^{M}(t+1) & = & \omega v_{i}^{M}(t)+\varphi_{1}r_{1}(p_{i}^{M}-x_{i}^{M}(t))+\\ & & \Phi \varphi_{2}r_{2}(p_{g}^{M}-x_{i}^{M}(t))+\left(1-\Phi \right)\varphi_{3}r_{3}(p_{g}^{S}-x_{i}^{M}(t)) \end{array}$$ (3)

$$\begin{array}{c} x_{i}^{M}\left(t+1\right)=x_{i}^{M}(t)+v_{i}^{M}\left(t\right) \end{array}$$ (4)

where *ϕ* is a migration factor, given by:

$$\begin{array}{c} \Phi =\{\begin{array}{cc} 0 & Gbest^{S}<Gbest^{M}\\ 0.5 & Gbest^{S}=Gbest^{M}\\ 1 & Gbest^{S}>Gbest^{M} \end{array} \end{array}$$ (5)

In Eq 5, *M* denotes the master swarm, and *S* the sub-swarm. *r*_3_ is a random number between 0 and 1 and *φ*_3_ is an acceleration constant. In the approach, the fittest particle among all (in both master and sub-swarms) gets the chance to guide the flight direction of the particles in the master swarm. Further the master swarm updates the particle states based on the both its own experience and that of the slave swarms.

A time varying inertia weight that changes exponentially over time is used in H-MCPSO, as in Eq 6 below, leading to constant sampling step and a smooth transition of the swarm from a global to more local search [17].

$$\begin{array}{c} \omega (c)=\frac{\omega_{max}}{e^{c}} \end{array}$$ (6)

$$\begin{array}{c} \Delta c=\frac{\left(10\omega_{max}\right)}{g_{max}} \end{array}$$ (7)

where *c* ∈ [0,(10*ω*_*max*_)] and *g*_*max*_ is the desire number of inertia weight change.

### Body Model

**Fig 2:**
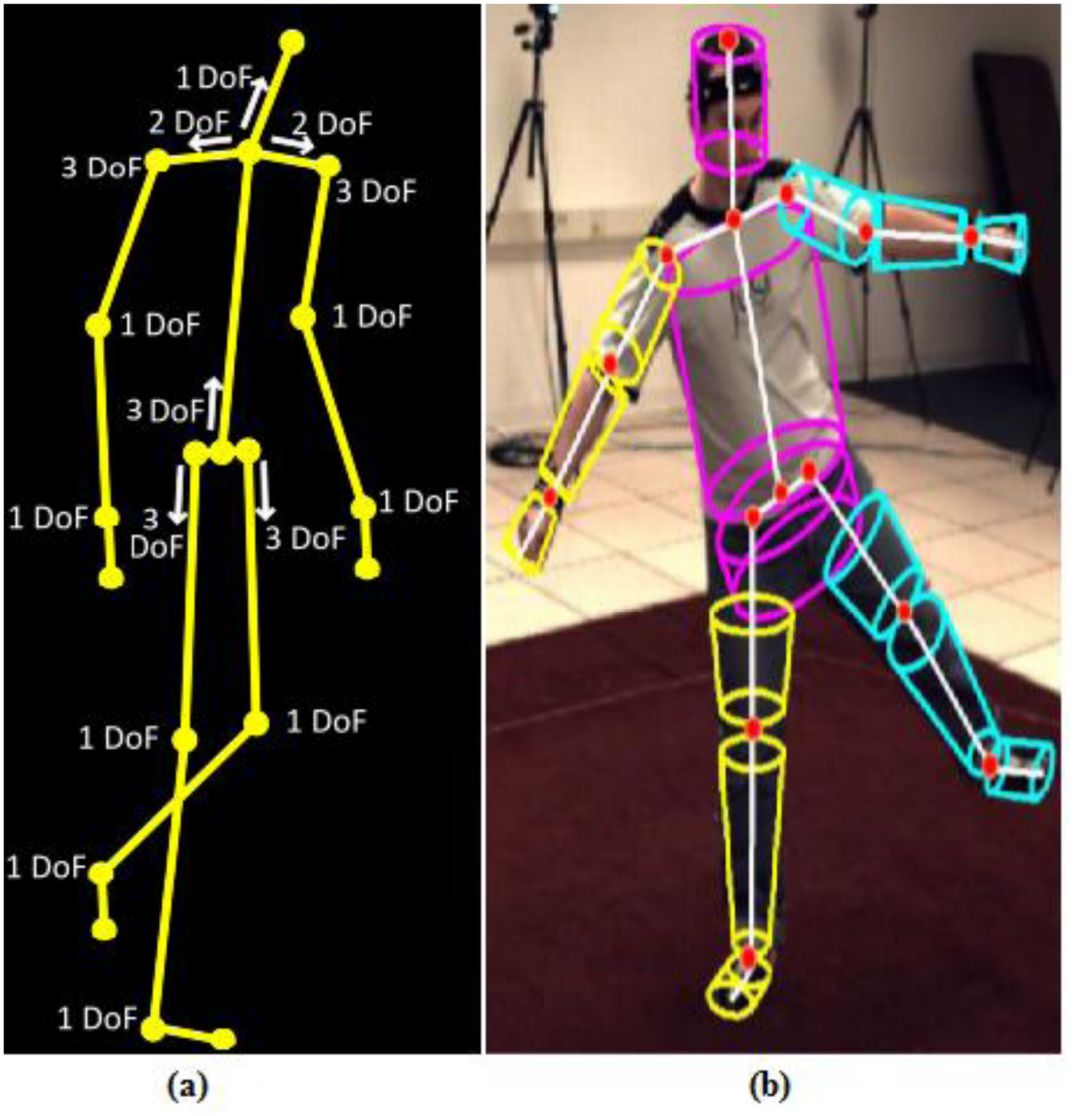
Parametrization of the skeleton. (a) The kinematic tree of the body model with respective number of DOF for all joints (b) The 3D body model with 15 body parts.

We have employed an articulated model similar to that in [36], as shown in Fig 2. The hips, shoulders and thorax are modeled as ball and socket joints (3DOF); the clavicles (2DOF), knees, ankles, elbows, wrist (1DOF) and head are assumed to be hinge joints (1DOF). The complete body model parameters comprise of 34 DOFs including the global position and orientation of the torso. The red spheres in Fig 2(a) are the joint locations where virtual markers are placed for computing 3D error. The complete body model, *X*, as a 34-dimensional vector, is as follows:

$$\begin{array}{c} X=\{\tau_{x},\tau_{y},\tau_{z},\theta_{x}^{1},\theta_{y}^{1},\theta_{z}^{1},\cdots ,\theta_{x}^{N},\theta_{y}^{N},\theta_{z}^{N}\} \end{array}$$ (8)

*X* in Eq 8 above comprises of two parts: *X* = (*X* ^*g*^, *X* ^*l*^). *X* ^*g*^ comprises of the position *τ*_*x*_, *τ*_*y*_, and *τ*_*z*_ and orientation $\theta_{x}^{1}$, $\theta_{y}^{1}$, $\theta_{z}^{1}$ of the pelvis. *X* ^*l*^ comprises of the rest of the parameters in *X*: torso (pelvis area and thorax with abdomen) (3DOF: $\theta_{x}^{2},\theta_{y}^{2},\theta_{z}^{2}$), left clavicle (2DOF: $\theta_{x}^{3},\theta_{y}^{3}$), left shoulder (3DOF: $\theta_{x}^{4},\theta_{y}^{4},\theta_{z}^{4}$), left elbow (1DOF: $\theta_{y}^{5}$), left wrist (1DOF: $\theta_{y}^{6}$), right clavicle (2DOF: $\theta_{x}^{7},\theta_{y}^{7}$), right shoulder (3DOF: $\theta_{x}^{8},\theta_{y}^{8},\theta_{z}^{8}$), right elbow (1DOF: $\theta_{y}^{9}$), right wrist (1DOF: $\theta_{y}^{10}$), head (1DOF: $\theta_{x}^{11}$), left hip (3DOF: $\theta_{x}^{12},\theta_{y}^{12},\theta_{z}^{12}$), left knee (1DOF: $\theta_{y}^{13}$), left feet (1DOF: $\theta_{y}^{14}$), right hip (3DOF: $\theta_{x}^{15},\theta_{y}^{15},\theta_{z}^{15}$), right knee (1DOF: $\theta_{y}^{16}$), right feet (1DOF: $\theta_{y}^{17}$).

### Fitness function

The primary goal of pose estimation is to compute the most likely model configuration *X* ^*t*^ at each time instant *t*. A fitness function evaluates how well a candidate pose hypothesis matches the observation, i.e. the images from all four views at each time instant. Ideally, the model should be stable enough to handle statistical variability of the image for various input sequences and subjects. In our work, the fitness function comprises of the edge fitness *f*_*e*_ and the foreground silhouette fitness *f*_*s*_. The overall fitness is defined as follows:

$$\begin{array}{c} f=f_{e}+f_{s} \end{array}$$ (9)

#### Edge-based Fitness

Due to depth ambiguity, silhouette information alone is insufficient to inform on the body configuration, even with perfect background subtraction, especially when the body parts are partially occluded. Image edges can be used to reduce depth ambiguity for model matching. Generally, image edges are invariant with environmental conditions such as color, cloths and lighting, etc. and therefore, they allow for localization of visible body parts. Edges feature is hence valuable for pose tracking [4, 37]. The edges in an observed image are detected by thresholding image gradients to obtain binary maps [4]. The binary edge image is then masked with the dilated silhouette to remove spurious edges in the background, following which it is blurred with a Gaussian kernel and rescaled to the range [0, 1] to produce an edge distance map. Gaussian blurring is then used to approximate a distance map. To compute the edge fitness *f*_*e*_ for a candidate pose, the edge map is sampled at discrete points along the visible edges of the candidate pose (as per [4]) and the following equation is then used to calculate the Sum of Squared Difference (SSD) between the edges in the map and the projected discrete points [4, 36].

$$\begin{array}{c} f_{e}=\sum^{s}(X,Z)=\frac{1}{N_{e}}\sum_{i=1}^{N_{e}}{\left(1-P_{i}^{e}\left(X,Z\right)\right)}^{2} \end{array}$$ (10)

In Eq 10 above, *X* represents the model points and *Z* the image from which the distance map is inferred. $P_{i}^{e}\left(X,Z\right)$ are the values of the edge distance map at the projected model points.

#### Silhouette-based fitness

Silhouette is considered as a strong cue for pose tracking [36–38] and it is insensitive to surface discrepancies such as texture and color variation. The silhouette fitness *f*_*s*_ measures the overlap between the observed and the projected silhouette. The observed silhouette images are acquired by performing a background subtraction from the original image. The projected silhouette is acquired by projecting the cylinders of the candidate pose into the respective view.

Shadows cast from the foreground subject onto the environment may be incorrectly classified as foreground [18]. To counter this problem, as in [36], each image pixel is modeled as a mixture of Gaussian distributions with mean *μ* and covariance *ρ*. Further, the foreground may also contain shadowed highlighted area caused by the moving subject. To counter this, the risk factor *δ* is added as follow:

$$\begin{array}{c} N(pix,\mu ,\rho )<\frac{1}{256\times 256\times 256\delta } \end{array}$$ (11)

**Fig 3:**
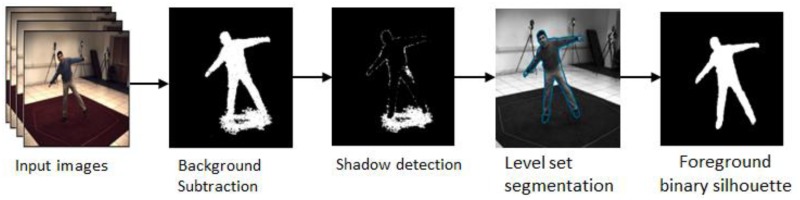
Graphical sketch of the enhanced silhouette extraction.

The highlighted area will be eliminated from the foreground as the values of *δ* increases. But, there is a possibility that a part of true foreground gets eliminated along with the highlighted area. As the foreground silhouette quality plays a major role in influencing the outcomes of tracking algorithm [17, 36, 37], we take further steps to improve the silhouette extraction, as shown in Fig 3.

Initially, *δ* in Eq 11 is removed to get the foreground information. Then, shadow detection and removal are performed by applying a deterministic non-model-based approach which is based on HSV color space. The computational complexity of this approach is relatively low and it is resilient to noise [39]. The decision process involved can be described as in the equation below:

$$\begin{array}{c} SP_{k}(x,y)=\{\begin{array}{cc} 1 & \omega \leq \frac{I_{k}^{V}(x,y)}{B_{k}^{V}(x,y)}\leq \varepsilon \wedge (I_{k}^{S}(x,y)-B_{k}^{S}(x,y))\\ & \leq \tau_{S}\wedge |I_{k}^{H}(x,y)-B_{k}^{H}(x,y)|\leq \tau_{H}\\ 0 & otherwise \end{array} \end{array}$$ (12)

where *I*_*k*_(*x*, *y*) denotes co-ordinate pixel value in the input image (frame k) and *B*_*k*_(*x*, *y*) is the co-ordinate pixel value in the background model. *ɛ* avoids those points where noise has changed the background slightly from being classified as shadows. The light intensity is defined by *ω*.

Finally, the silhouette is refined by using the level set approach in [40]. In this approach, region-based segmentation energy is re-formulated in a local manner, enabling it to segment objects having heterogeneous profiles.

From the above silhouette extraction process, a binary foreground silhouette map is obtained. The modeling of constraints is done such that the silhouette of the body model projects within the image silhouette. For computational efficiency, only a discrete number of points within the limbs are checked [36]. The computation of SSD between the projected point and the silhouette is done as per the following equation [4, 36]:

$$\begin{array}{c} f_{s}=\sum^{s}(X,Z)=\frac{1}{N_{s}}\sum_{i=1}^{N_{s}}{\left(1-P_{i}^{s}(X,Z)\right)}^{2} \end{array}$$ (13)

where $P_{i}^{s}\left(X,Z\right)$ denotes the values of foreground pixel map taken from the interior of the 3D body model at the N sampling points.

To further strengthen the effectiveness of our silhouette detection module, in H-MCPSO, we incorporate a bi-directional detection method used in [36]. The bi-directional silhouette calculates how much of the projected silhouette overlaps the observed, as well as how much of the observed silhouette overlaps with the projected. This approach avoids unreasonably high fitness values for poses with overlapping limbs [36, 41].

### H-MCPSO algorithm for pose tracking

In tracking applications, the data of concern is temporal in nature. This allows the use of a prior estimation to cut down the search space required to make a new estimation. From the Bayes' view, the pose tracking problem can be formulate as:

$$\begin{array}{c} p(x_{t}|z_{t})\propto p(z_{t}|x_{t})p(x_{t}|x_{t-1}) \end{array}$$ (14)

where *x*_*t*_ and *z*_*t*_ are the state vector and the observation respectively at time *t*. The basic problem for 3D human pose estimation is the determination of the conditional distribution *p*(*x*_*t*_∣*x*_*t* − 1_). The process depicted in Eq 14, assume a first-order Markov process [36, 42]. In the subsections to follow, we described the 3 main stages—initialization, hierarchical pose estimation, and next-frame propagation—involved in our solution for this.

#### Initialization

The main aim of pose initialization is to recover the initial 3D pose of the subject. In general, it is an intractable problem, as no temporal information can be used. The problem, in most approaches, is reduced either by adjusting the model to the first frame manually, or simply by making assumption about the subjects initial pose (special start pose) (e.g. [6, 43]). On the other hand, in this work, we run the H-MCPSO algorithm on the very first frame itself in order to derive the initial pose.

**Fig 4:**
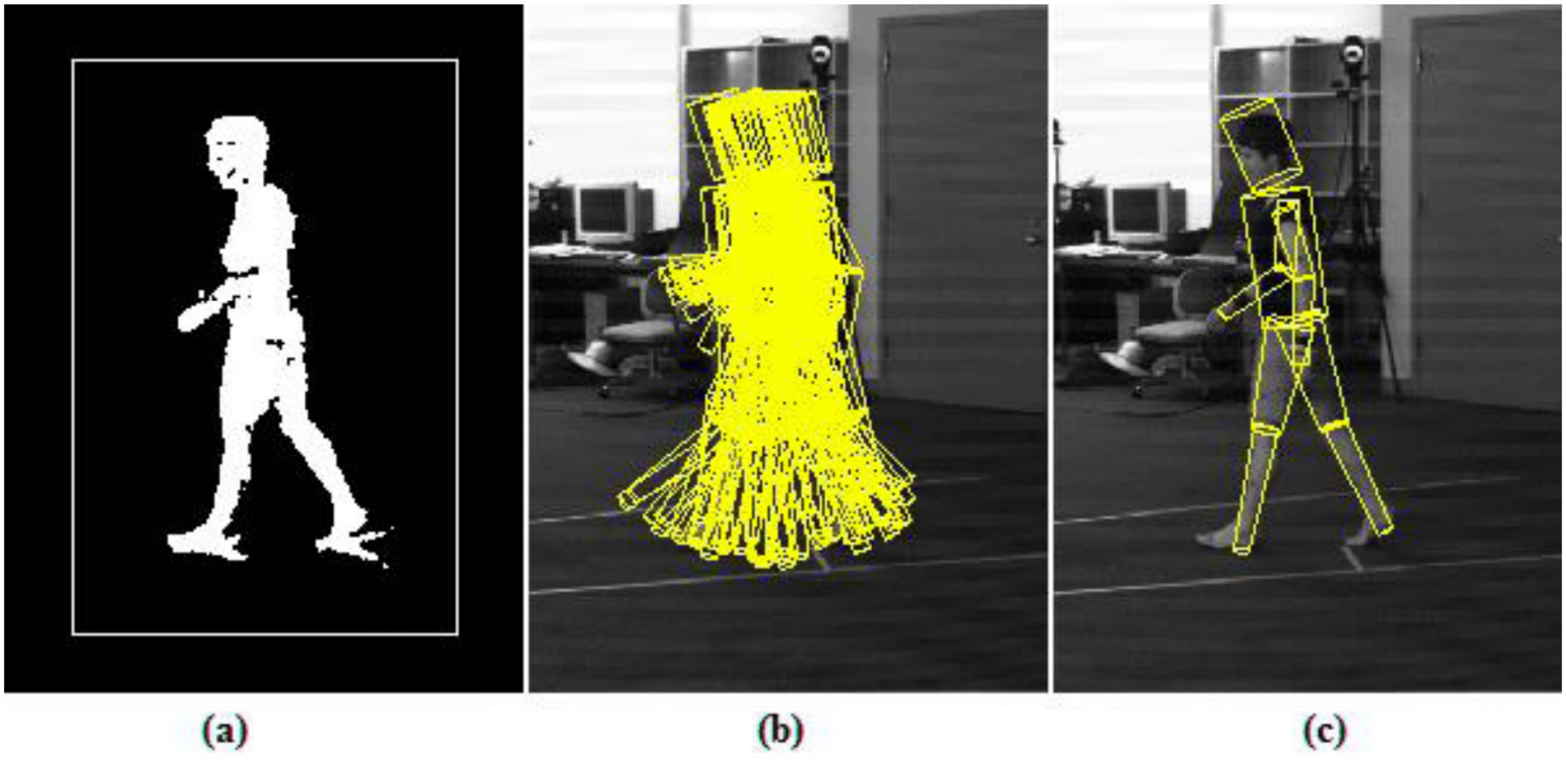
Automatic initialization. (a) constrained search space (b) initial set of particles is randomly distributed around the center of the truncated pose (c) The final correctly initialized pose after few iterations.

Fig 4, illustrates the automatic initialization, where the H-MCPSO algorithm, initialized by sampling from a random distribution centered at the truncated pose, consistently found the correct position and orientation of the person in the initial frame.

#### Hierarchical pose estimation

**Fig 5:**
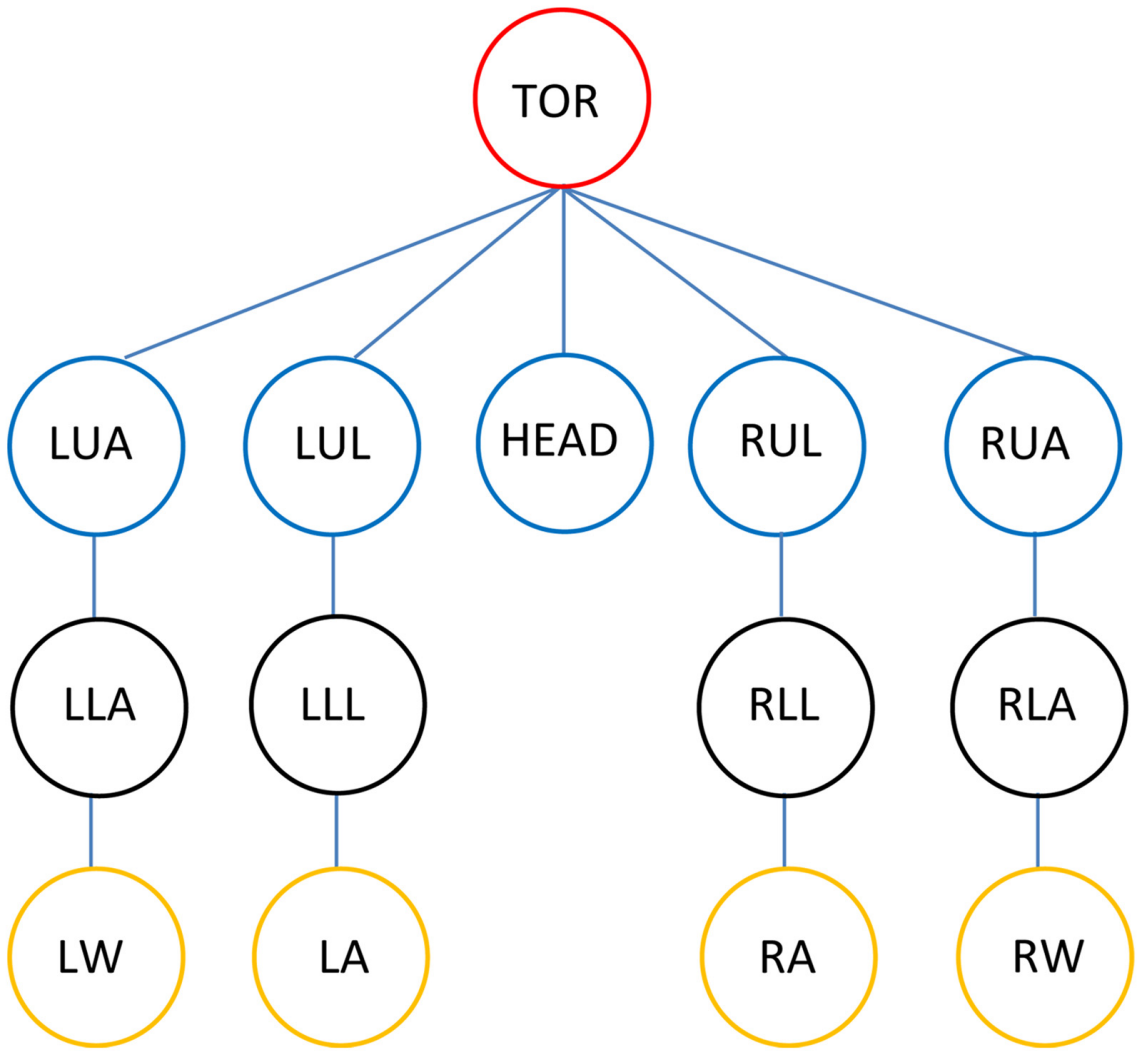
Kinematic tree. Standard Kinematic tree representation of human model with five open kinematic chains.

Pose estimation with H-MCPSO is performed by maximizing the fitness function for every frame. In order to reduce the search complexity, the pose search process is performed in a hierarchical manner. We divide the search space into six different sub-spaces and correspondingly execute the optimization in 6 hierarchical steps. The six steps are: the global position and orientation of the root followed by, torso and head, and finally, the branches corresponding to the limbs (as illustrates in Fig 5), each of which is optimized independently. The standard Kinematic tree representation of human model with five open kinematic chains is illustrated in Fig 5, where LUA, LLA and LW define the left upper arm, left lower arm and left wrist respectively; similarly LUL, LLL and LA represent the left upper leg, left lower leg and left ankle respectively; similar representation is on right side for the right body parts and TOR define the torso of the body.

In order to reduce errors accumulating as we traverse the hierarchy, we employed a soft partitioning approach. Previously optimized parameters which are positioned higher in the tree are allowed some variation in the subsequent optimization stages. For instance, when optimizing the torso and the head, the global body position and orientation of body parameters are allowed some variation. Similarly, when any of the limbs is being optimized, the torso node is allowed a little variation in order to improve the overall fitness.

Further, to obtain reliable and robust pose estimation results, we enforce 2 motion constraints. Firstly, the angles must follow the anatomical joint limits (hard limits). In our H-MCPSO algorithm, angle limitation can be applied easily by confining the flight of the particles. Secondly, body limbs do not inter-penetrate.

In all, the H-MCPSO algorithm is as per the algorithm below:

**Table 1:** Algorithm 1.

Initialize master swarm and sub-swarms;
**for** *each particle in whole population* **do**
Initialize position and velocity;
Evaluate fitness;
**end**
**repeat**
**for** *each sub-swarm* **do**
Evolve subswarm;
**end**
Barrier synchronization waits until all processes to finish;
Insert the fittest particles from each of the subswarms into master swarm;
Evolve master swarm;
Record best particle in master swarm;
**until** *maximum iteration or termination condition is true*;

### Next-frame propagation

As previously indicated, H-MCPSO utilizes only limited amount of inter-frames information and does not include any motion model. Upon estimation of pose in a particular frame, the swarm of particles for the incoming frame is initialized by sampling a Gaussian distribution centered in the best estimate for the current frame. The Gaussian covariance attribute is set to a minimal value—0.01 in our experiments—for all joints in order to enhance temporal consistency.

## Experimental results and analysis

Experiments were conducted using the publically available HumanEva-II [36] and the Brown dataset [37], as they are more frequently reported datasets in articulated human motion tracking research literature.

In first set of experiments, two video sequences, “S2” and “S4”, from HumanEva-II [36] dataset were used to evaluate the performance of the proposed algorithm. The HumanEva-II contains two subject sequences (“S2”, “S4”) and the sequence was captured by four synchronized color cameras in a studio environment with 656 × 490 resolutions at 60Hz. The human model used in this case has 34 DOF (as described in early section).

In second set of experiments, the Brown database consists of a walking sequence (“Lee Walk”) captured by four synchronized grey scale cameras in a studio environment with 644 × 488 resolutions at 60Hz (original) were used. In this case, the human body model has 31 DOF. This body model is different from HumanEva-II is that it has one degree of freedom less for the wrists and ankles. Further, the torso joint has one additional degree of freedom.

In both datasets, the recording of the video was done on a subject wearing reflective markers and captured using a motion capture system. The position of reflective markers on the subject corresponds to that on the 3D model. Hence the distances of projected virtual points to their ground truth positions can be treated as errors during the tracking process. As in [36], the error metric is defined as the average absolute distance between the actual position, *x*, and the corresponding estimated position, $\hat{x}$, for each marker, as per the equation below:

$$\begin{array}{c} d(x,\hat{x})=\frac{1}{n}\sum_{i=1}^{n}∥x_{i}-{\hat{x}}_{i}∥ \end{array}$$ (15)

Eq 15 gives an error measure for a single frame of the sequence. The tracking error of the whole sequence is calculated by averaging the error measures for all the frames.

The proposed algorithm was implemented using the code made available by the authors of [36]. For comparison purpose, we implemented and tested as well two other state-of-the-art algorithms, namely the APF and HPSO, both using the same code base.

### H-MCPSO algorithm parameter setting

The implementations for each of the algorithms—H-MCPSO, HPSO, and APF—were done using Matlab and tested on a desktop computer running Windows on a dual-core 3.20GHz processor. The total number of likelihood evaluations for each of the algorithms was fixed to be at 3600 per frames.

For H-MCPSO, we utilized as well the Matlab parallel computing toolbox to allow for some parallelism in the execution of the swarms. The number of sub-swarms was set at 3 and the population at 20, split among the sub-swarms—5 in each sub-swarm and 5 in the master swarm. Acceleration parameters were set at *φ*_1_ = *φ*_2_ = 2.05, *φ*_3_ = 2.0 and *ω*_*max*_ = 2.0 and *ω*_*min*_ = 0.1. The maximum number of iteration was set at 30.

For the HPSO, the parameters were as in [17]. As with H-MCPSO, we ran HPSO using 6 hierarchical steps. The APF algorithm was ran with 600 particles with 6 annealed layers and the APF algorithm implemented code is taken from [36].

The parameter settings above were maintained across all the test sequences, i.e., no specific tuning was made for any particular movement or subject. All the tested algorithms are highly stochastic in nature and produce different results for different runs for the same configuration of parameters. So, each experiment was run 5 times for each of the video sequences to get a measure of the performance consistency and repeatability.

### Quantitative and visual results

**Fig 6:**
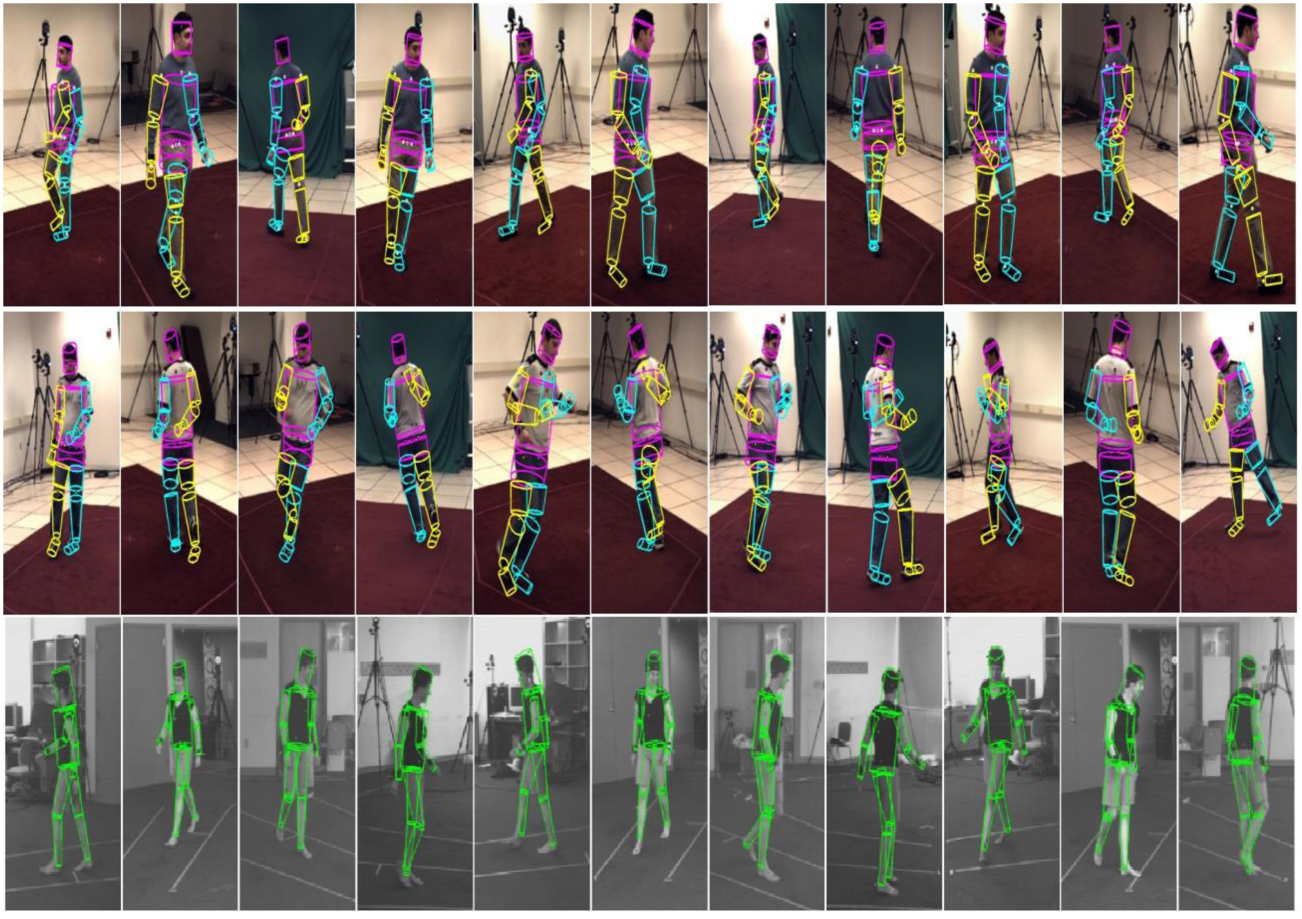
Visual tracking results. H-MCPSO tracking output on the HumanEva-II, subject “S2” (top row), Subject “S4” (middle row) and “Lee Walk” (bottom row) using H-MCPSO. Top row shows every 25th tracked frames; middle row shows every 50th tracked frames (starting from the 350th frame) and bottom row sows the every 10th tracked frames of the first 100 frames in the “Lee Walk” sequence.

Fig 6, (S1 video and S2 video) shows the visual outcome of the H-MCPSO run. The jogging activity is error-prone due to the rapid limb movements involved. The outcome however indicates that H-MCPSO is insensitive to the accumulation of error.

**Fig 7:**
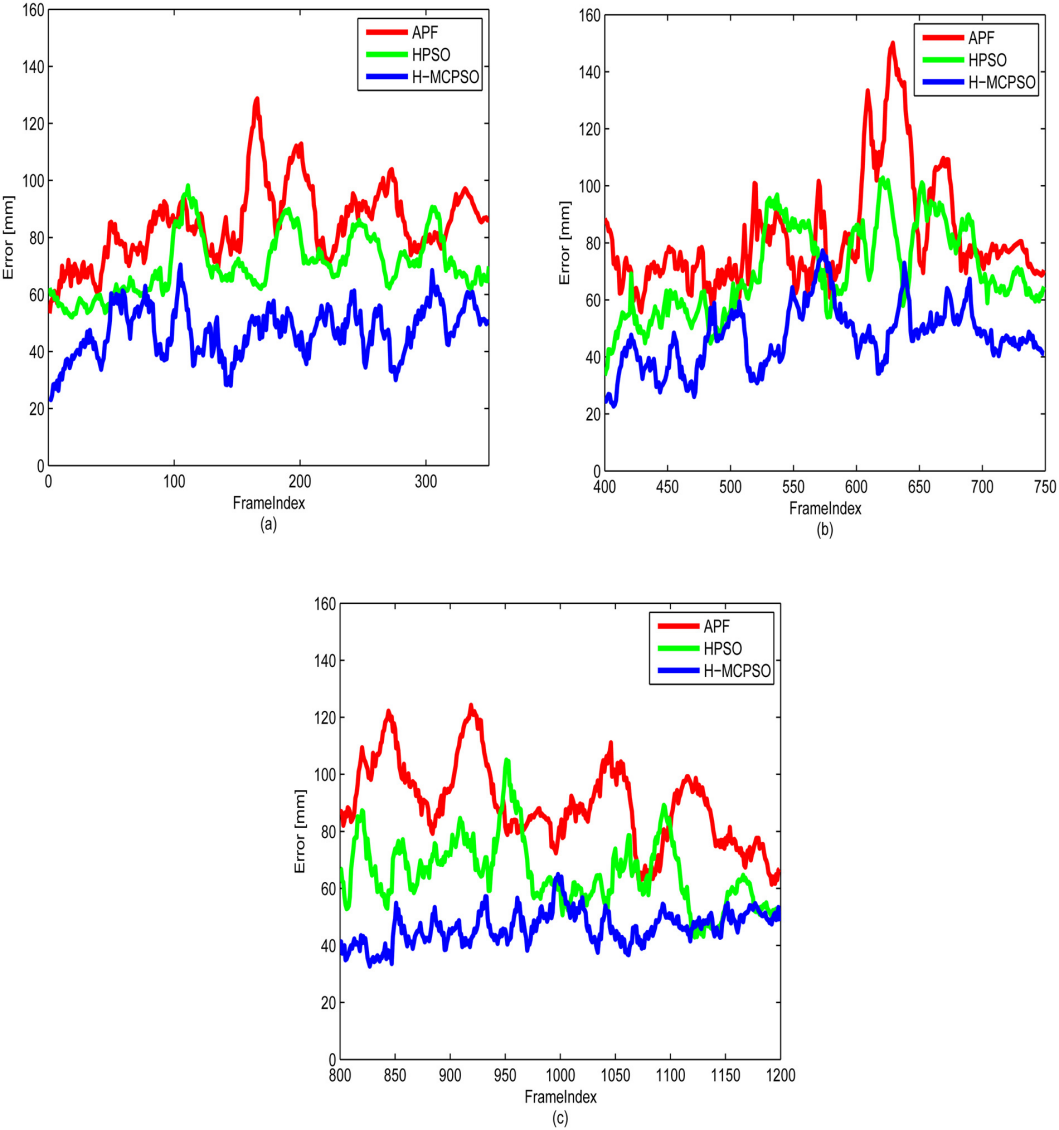
Tracking errors with respect to every video frame on the HumanEva-II, subject “S2”. (a) Walking (b) Jogging (c) Balancing.

Fig 7 shows the average 3D tracking errors obtained by different implemented approaches. Table 2 shows the 3D tracking error in greater details indicating the difference in values between the ground-truth joints values and the pose estimated in each frame, averaged over 5 runs on HumanEva-II subject S2 for walking, jogging and balance activity. The experimental results clearly show that the H-MCPSO tracking performance compares favorably to the performance of APF and HPSO. We believe that the better performance in H-MCPSO is a consequence of diversity maintained in the multi-swarm evolutionary process. Further, we note that the error rate for the APF algorithm are higher than that reported in [36], even though the code we used is that provided by the authors of [36]. Similar disparity has been noted in other publications [7, 17, 44].

**Table 2:** Absolute 3D error calculated for the HumanEva-II sequence S2 and S4 (60Hz) (5 run).

	S2			S4		
Algorithm	Walk	Jog	Balance	Walk	Jog	Balance
APF	84±4mm	92±9mm	98±4mm	64±8mm	94±2mm	82±3mm
HPSO	62±6mm	72±3mm	68±12mm	52±11mm	68±12mm	56±4mm
H-MCPSO	48±4mm	52±6mm	42±6mm	38±2mm	50±5mm	44±3mm

### Accuracy

The tracking results in Table 2 suggest that the proposed H-MCPSO algorithm is able to estimate the pose more accurately and consistently as compared to HPSO and APF for all the considered sequences. All the tested algorithms utilized the same likelihood function.

**Fig 8:**
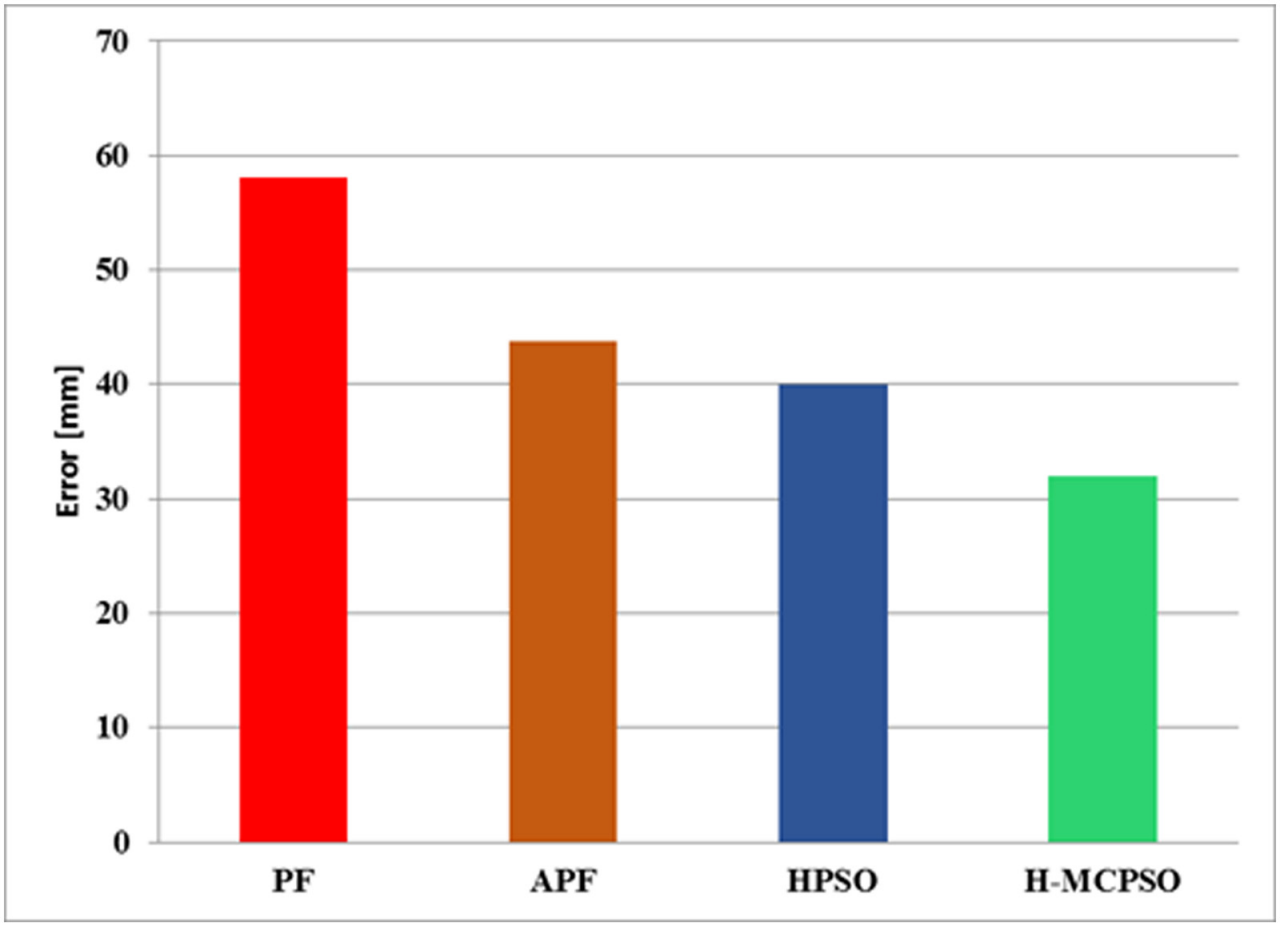
Comparison of 3D error of various algorithms for Lee Walk Sequence. Frame rate: (60Hz).

We tried as well to compare the performance of H-MCPSO to particle filter (PF) with the same dataset and the same body model. However, since our body model allows large mobility, that is degree of freedom, in the limbs, the PF algorithm often produced very poor tracking results or required large number of particles, leading to impractically long processing time on our machine. We did however manage to run the algorithm on the Lee Walk sequence with 31 DOF human body model, obtaining the tracking error results shown in Fig 8.

### Self-recovery from wrong estimates

**Fig 9:**
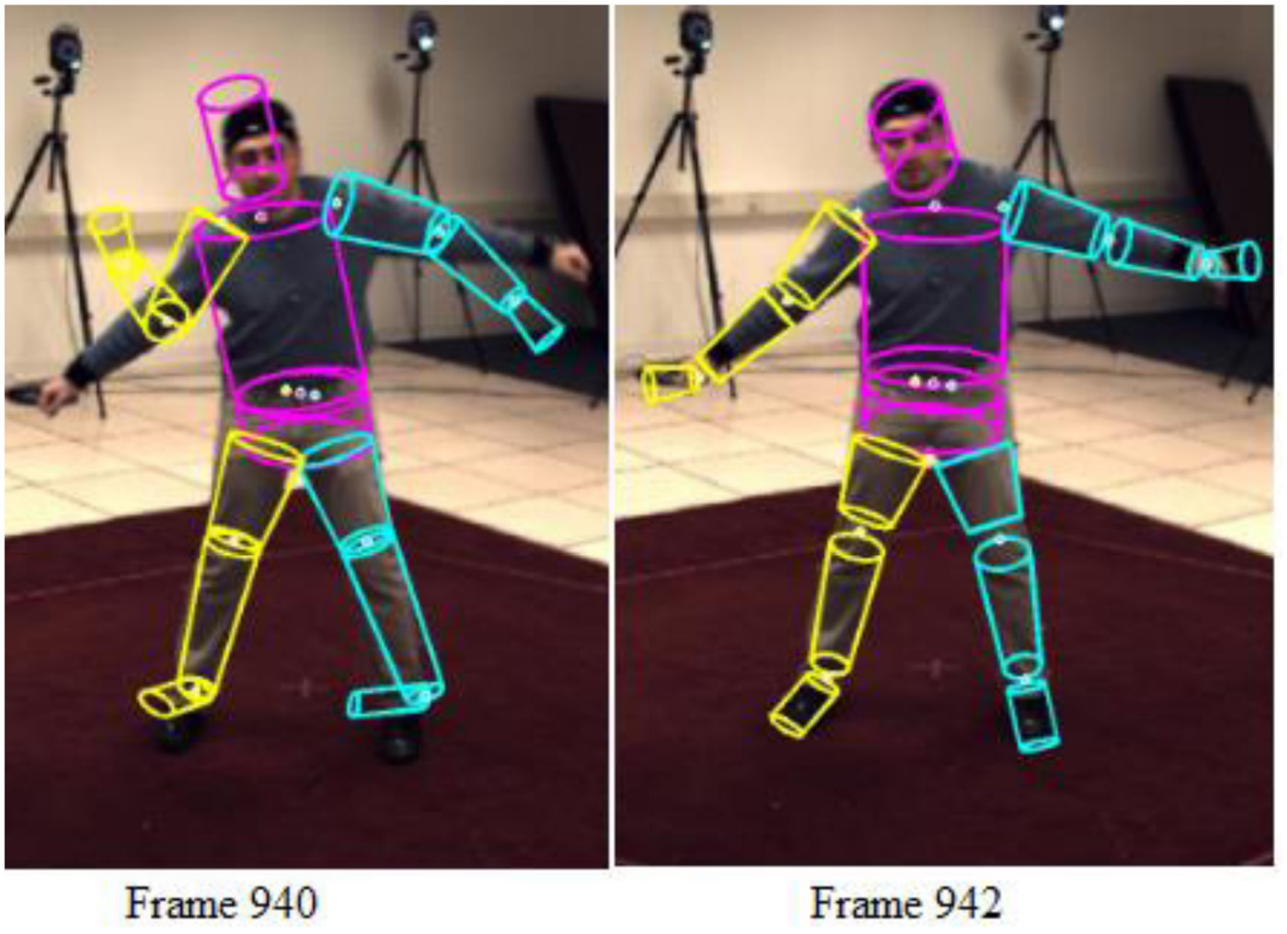
illustration of self-recovery from temporary tracking failures.

Occasional wrong estimates may be due to several factors, pertinent among which includes noisy silhouette segmentation and self-occlusion leading to ambiguous poses. However, H-MCPSO has shown a systematic ability to self-recover from temporary tracking failures within a few frames. For example, Fig 9 shows the algorithm losing track of multiple limbs (hands and legs) in frame number 940. Within a few frames however, by frame 942, the tracking recovered.

In comparison, we noticed that the tracking failures in the other implemented algorithms, especially PF and APF, are more common and the lost tracking tend to be unrecoverable, i.e., the estimates would diverge. The ability of H-MCPSO to self-recover is very likely due to the use of multiple swarms, enabling the exploration of sufficiently large region of the search space with a limited number of particles.

### Varying the number of particles

We have evaluated the H-MCPSO performance by varying the number of particles. However, to keep the computational time feasible on our hardware, the range of *N* was limited. The evaluation was done with 20, 40 and 60 particles over five trials. The Table 3 shows the results. As predicted, we noticed that the accuracy and consistency has improved as the value of *N* increased; the computational time, however, increased as well.

**Table 3:** H-MCPSO's 3D error in mm for the HumanEva-II on S2 sequence (60Hz) with varying number of particles and likelihood evaluation.

Algorithm	Walk	Jog	Balance
H-MCPSO (20 particles)	48±4mm	52±6mm	42±6mm
H-MCPSO (40 particles)	38±6mm	42±4mm	36±10mm
H-MCPSO (60 particles)	34±4mm	40±6mm	34±8mm

The more the number of particles, the greater the number of likelihood evaluations per frame. 20 particles required 3600 likelihood evaluations, 40 particles required 7200 and 60 particles required 10,800 evaluations per frame. Extrapolating the experiments to evaluate the limiting value of *N* beyond which no significant benefit happens is beyond the scope of our hardware.

### Varying the number of sub-swarms

To evaluate the benefit of the sub-swarms in H-MCPSO algorithm, we tested the performance using different number of sub-swarms with fixed number of particles. We ran H-MCPSO with 2, 3, 4 and 5 sub-swarms. Using more number of sub-swarms with fixed number of particles (20 particles) helps to prevent the tracker from losing track and improves the performance. This is because more number of swarms able to explore more search space and also able to maintain diversity therefore it produces more promising results than single swarm PSO. The results obtained are tabulated in Table 4. However, increasing the number of sub-swarms seems to have minimal benefit for the balancing activity; it may be due to lesser movement in the body limbs.

**Table 4:** H-MCPSO's 3D error for the HumanEva-II on S2 sequence (60Hz) by varying number of sub-swarms with fixed number of particles (20).

Algorithm	Walk	Jog	Balance
H-MCPSO (5 sub-swarms)	44±8mm	47±10mm	41±11mm
H-MCPSO (4 sub-swarms)	48±10mm	50±11mm	41±8mm
H-MCPSO (3 sub-swarms)	48±4mm	52±6mm	42±6mm
H-MCPSO (2 sub-swarms)	58±9mm	66±7mm	46±15mm

### Hierarchical vs. non hierarchical

We compare as well the performance of H-MCPSO with non-hierarchical PSO and non-hierarchical multi-swarm PSO (MCPSO), using the HumanEva-II S2 sequence at 60Hz. The complete setting remained the same except that the number of inertia changes (*g*_*max*_) was set to 180 for PSO and MCPSO. Table 5 shows that performances of PSO and MCPSO were both comparable to that for the APF algorithm. The hierarchical versions of both approaches produced better tracking results.

**Table 5:** Performance of hierarchical and non-hierarchical on HumanEva-II sequence S2.

Algorithm	Walk	Jog	Balance
APF	84±4mm	92±9mm	98±4mm
PSO	72±0mm	78±6mm	66±6mm
MCPSO	60±2mm	70±8mm	60±6mm
HPSO	62±6mm	72±2mm	68±12mm
H-MCPSO	48±4mm	52±6mm	42±6mm

### Computation time

Computation time is a major concern in pose tracking. Generally, it takes from seconds to minutes, for a Matlab implementation, to estimate the pose in a single frame [17, 36, 37]. This means that tracking an entire sequence may take hours. However, the computation time vastly depends on the number likelihood evaluation and the form of model rendering. To compare computation times, we have ensured the same number of likelihood evaluation for all implemented approaches—that is 3600 per frame. The number of likelihood evaluations for each algorithms is calculated as follows:

- APF: 600 particles × 6 layers = 3600 per frame.
- PSO and MCPSO: 20 particles × 180 inertia change = 3600 per frame.
- HPSO: 10 particles × 60 iteration × 6 hierarchical steps = 3600 per frame.
- H-MCPSO: 20 particles × 30 iteration × 6 hierarchical steps = 3600 per frame.

Run-time of various algorithms on whole body human tracking are shown in Table 6. H-MCPSO took an average of 120.8 secs per frame with bi-directional silhouette combined with edge (BiS+E), while with the simple standard silhouette (one-sided) with edge (S+E), it took 48.6 secs. HPSO algorithm took about 108.5 secs with (BiS+E) and 42.4 secs with (S+E). As shown in Table 6, the APF algorithm required the heaviest execution time.

**Table 6:** Computation time of various algorithms. FE: Fitness Evaluation.

Algorithm	FE	BiS+E	S+E	BiS+S(per FE)	S+E(per FE)
APF	3600	392.2sec	88.8sec	108.94ms	24.66ms
PSO	3600	192.5sec	76.2sec	53.47ms	21.16ms
HPSO	3600	108.5sec	42.4sec	30.13ms	11.77ms
MCPSO	3600	204.2sec	82.4sec	57.83ms	22.88ms
H-MCPSO	3600	120.8sec	48.6sec	33.55ms	13.5ms

The execution time for the proposed H-MCPSO algorithm was a little longer than that for HPSO. The longer execution time is to be expected as the H-MCPSO evolves multiple swarm. However, the fact that it was able to obtain significantly better tracking results at the cost of slightly longer execution time points to its practicality.

## Conclusion and future work

The most challenging issue in model-based markerless articulated human body motion tracking is the high dimensionality of the parametric search space involved. The solution to this problem requires a search strategy that can efficiently explore a wide region of the search space. The proposed H-MCPSO algorithm shows promising results. Qualitative and quantitative comparisons between H-MCPSO algorithm and currently extensively used algorithms, especially HPSO and APF, shows that H-MCPSO gives better tracking performance. H-MCPSO effectively escapes from local maxima by utilizing multiple swarms. Further, the soft partitioning approach deployed by the algorithm proved to be effective in overcoming error accumulation.

We notice that in our experiments, tracking was always lost only temporarily and its recovery attained systematically after one or a few frames. Wrong pose estimates are probably due to poor silhouette segmentation in some cameras and self-occlusion. The body model, which has been incorporated in our experiment, is composed only of cylinders [36, 37], presenting a front-back ambiguity for poses in which all the skeleton segments lie in a plane. The problem can be resolved by using non-symmetric surface models as presented in [45].

The H-MCPSO method we have presented in the paper contributes to research in markerless human motion tracking system. It can potentially leads to a low-cost yet robust tracking solution. The potential benefits in various domains will be tremendous, especially in biomedical domain—our prime application area of interest. We intend to apply the technique to monitor the stroke rehabilitation progress of patients in real clinical context. Future research includes investigation of dimensional reduction techniques to reduce the parametric search space so that tracking accuracy and robustness of algorithm can be further improved. Another possible interesting improvement could be the use of increasingly popular 3D sensors, such as low-cost depth cameras, instead of specialized multi-camera setup, for easier acquisition of image; this can dramatically extend the usability of our approach.

## Supporting Information

S1 video Lee Walk sequence. (AVI)

S2 video HumanEva-II S2 sequence. (AVI)
